# Absence of Tissue-Sparing Effects in Partial Proton FLASH Irradiation in Murine Intestine

**DOI:** 10.3390/cancers15082269

**Published:** 2023-04-13

**Authors:** Qixian Zhang, Leo E. Gerweck, Ethan Cascio, Liqun Gu, Qingyuan Yang, Xinyue Dong, Peigen Huang, Alejandro Bertolet, Konrad Pawel Nesteruk, Wonmo Sung, Aimee L. McNamara, Jan Schuemann

**Affiliations:** Physics Division, Department of Radiation Oncology, Massachusetts General Hospital & Harvard Medical School, Boston, MA 02129, USA

**Keywords:** FLASH effect, proton irradiation, intestine, lymphocyte depletion

## Abstract

**Simple Summary:**

The normal tissue-sparing effect of ultra-high dose rate irradiation (i.e., FLASH effect) has been widely reported. However, our data suggest that not all irradiations with a dose rate above 40 Gy/s can confer benefits. We found that partial abdominal FLASH proton irradiation neither improved survival nor preserved circulating lymphocytes. These findings highlight the necessity to understand the conditions that induce the FLASH effect, for successful clinical translation.

**Abstract:**

Ultra-high dose rate irradiation has been reported to protect normal tissues more than conventional dose rate irradiation. This tissue sparing has been termed the FLASH effect. We investigated the FLASH effect of proton irradiation on the intestine as well as the hypothesis that lymphocyte depletion is a cause of the FLASH effect. A 16 × 12 mm^2^ elliptical field with a dose rate of ~120 Gy/s was provided by a 228 MeV proton pencil beam. Partial abdominal irradiation was delivered to C57BL/6j and immunodeficient Rag1^−/−^/C57 mice. Proliferating crypt cells were counted at 2 days post exposure, and the thickness of the muscularis externa was measured at 280 days following irradiation. FLASH irradiation did not reduce the morbidity or mortality of conventional irradiation in either strain of mice; in fact, a tendency for worse survival in FLASH-irradiated mice was observed. There were no significant differences in lymphocyte numbers between FLASH and conventional-dose-rate mice. A similar number of proliferating crypt cells and a similar thickness of the muscularis externa following FLASH and conventional dose rate irradiation were observed. Partial abdominal FLASH proton irradiation at 120 Gy/s did not spare normal intestinal tissue, and no difference in lymphocyte depletion was observed. This study suggests that the effect of FLASH irradiation may depend on multiple factors, and in some cases dose rates of over 100 Gy/s do not induce a FLASH effect and can even result in worse outcomes.

## 1. Introduction

Since 2014 [1], reports of a normal-tissue-sparing effect of ultra-high dose rate irradiation (i.e., FLASH effect) has gained attention. The FLASH effect has most commonly been observed following irradiation at dose rates >40 Gy/s. Several mechanisms have been suggested to explain the dose-rate-dependent differential response in healthy tissue [2,3,4,5,6]. One prevailing hypothesis is that the FLASH effect is related to the fast consumption of oxygen during the short-lasting irradiation period. As early as 1959, Dewey et al. reported relatively lower radiosensitivity in Serratia marcescens when 100–200 Gy of X-ray radiation was delivered under the condition of 1% oxygen within 2 microseconds [7]. They attributed this phenomenon to the depletion of oxygen. The ultra-high-dose-rate-dependent increase in cell survival was confirmed in Escherichia coli B/r [8] and mammalian cells [9,10]. In in vivo studies, Hornsey et al. showed that for mice breathing oxygen during whole-body irradiation, the fraction of mice surviving irradiation increased as the dose rate increased from 60 Gy/min to 1000–5000 Gy/min. The effectiveness of irradiation did not vary with the dose rate when the mice breathed nitrogen during exposure [11].

Another hypothesis is that the number of circulating lymphocytes is much higher following FLASH vs. conventional irradiation, as FLASH irradiation is completed within an extremely short time. In theory, even though the lymphocytes inside the FLASH field receive a higher dose from FLASH, the small volume of exposed blood will spare more cells than conventional dose rate (CDR) irradiation (where the blood keeps cycling through the radiation field) does. Researchers at the Cleveland Medical Center provided a simplified model to calculate the cell-killing potency of diverse dose rate irradiations [6]. For FLASH irradiation, the exposure time t is much smaller than the blood circulation time T for one cycle. Thus, the blood flow throughout the treatment during the irradiation period t is negligible. For CDR irradiations, the T value of adult mice is usually less than the exposure time t. Then, the percentage of killed lymphocytes can be calculated from the accumulated volume and dose. However, Venkatesulu demonstrated more severe lymphocyte depletion following cardiac and splenic FLASH irradiation at a dose rate of 35 Gy/s [12]. More experimental studies of the potential impact of FLASH irradiation on the immune system need to be conducted for different tissues.

In recent years, FLASH photon or electron irradiation has been shown to reduce the damage to the lungs [1], gastrointestinal tract [13,14], brain [15,16,17,18,19], and skin of mice, as well as the skin of mini pigs [20,21,22]. Most of the studies utilized beams generated by modified accelerators. It has also been reported that a single dose of 15 Gy FLASH irradiation to the skin only caused a grade 1 reaction, without compromising tumor control in a human patient [23]. However, an update of this report suggested that a single dose of 15 Gy electron irradiation at a dose rate of 166 Gy/s generated similar acute and late skin responses compared to conventional irradiation [24]. 

Several groups have successfully established preclinical proton FLASH irradiation platforms and observed the proton FLASH effect. Researchers at the Institute Curie achieved a proton field of 12 × 12 mm^2^ through a single-scattering system with a ridge filter. The platform delivered protons at a dose rate that exceeded 40 Gy/s [25]. A group at the University of Pennsylvania reported minimal acute and late intestinal reactions after FLASH proton irradiation at a dose rate of 78 ± 9 Gy/s [13]. Cunningham et al. observed reduced leg contraction and skin injury with FLASH proton irradiation [26].

Our team has established a double-scattering system for proton research which offers an elliptical field of 16 × 12 mm^2^. Preliminary results indicate abdominal tissue protection with a dose rate of ~120 Gy/s [27]. Here, we report the completed study of abdominal tissue-sparing effects of FLASH proton irradiation with high statistical power and studies on circulating-lymphocyte depletion.

## 2. Materials and Methods

### 2.1. Animals

A total of 161 female C57BL/6j mice aged 10 to 12 weeks old were purchased from the Jackson Laboratory. All mice were housed in the Cox 7 animal facility holding room at the Massachusetts General Hospital (MGH) for at least one week prior to irradiation. The mice were randomly divided into different cohorts for FLASH irradiation or conventional dose rate irradiation. In addition, a total of 63 female Rag1^−/−^/C57 mice aged 8 to 10 weeks old with different body weights (averages of 18.8 g and 24.5 g, obtained from the Cox 7 facility) were used to investigate the relationship between FLASH irradiation and lymphocyte depletion. All mice were maintained in micro-isolation caging (5 animals per cage), fed sterile pelleted chow, and given acidified sterile water ad libitum. All animal care and procedures were performed in accordance with the Public Health Service Policy on Humane Care of Laboratory Animals and approved by the IACUC (Institutional Animal Care and Use Committee) at the MGH. 

### 2.2. Irradiation Procedure

Proton irradiations were performed at the experimental beamline of the Francis H. Burr Proton Center at the MGH. The proton FLASH irradiation platform has been previously described [27]. In short, the beamline is able to provide a 16 × 12 mm^2^ elliptical homogenous field for a 228 MeV double-scattered beam at a dose rate of ~120 Gy/s. The beam went through two scatterers, an aperture, and a ~4 cm polymethyl methacrylate (PMMA) block before reaching the mouse. Before irradiation, the dosimetry was verified using EBT3 Gafchronic film, a thimble chamber, a thin-gap parallel-plate ion chamber, a Faraday Cup, and Monte Carlo Simulation. 

The position of the radiation field in the mouse was confirmed using CT and MRI images taken prior to irradiation of a sample of 3–5 mice that were constrained in the same position on a custom mouse holder for irradiation. According to the scanning images, the field covered about 60% of the whole abdominal volume. The platform was able to irradiate two mice at a time, with the second mouse downstream of the first and with a ~3 cm air gap. The dose difference between the two positions was 16%, which means the two mice belonged to two dose cohorts. The mice were placed in the entry plateau region of a proton beam.

The mice were anesthetized with a mixture of Ketamine (100 mg/kg) and Xylazine (10 mg/kg) and restrained on a specially designed plastic holder by holding their forelegs with two clips. The rear end of each mouse’s body was firmly pressed against the vertical plastic plate to ensure reproducibility of the positioning. The mice received a single dose of 14 Gy, 15.1 Gy, 16 Gy, 16.2 Gy, 17 Gy, 17.5 Gy, or 18 Gy, with either a FLASH dose rate (ranging from 112 Gy/s to 128 Gy/s) or a conventional dose rate (ranging from 3.2 Gy/min to 24.0 Gy/min). FLASH and CDR irradiations were performed alternately, switching modalities between each mouse. For conventional dose rate irradiation, we kept the exposure time (~ 0.60 min) the same for all dose levels to produce similar irradiation-time structures for circulating lymphocytes. 

### 2.3. Blood Sampling

Blood samples were collected only in the first experiment. Fifteen mice from each group were randomly divided into three sets to avoid frequent blood draws from the same mice within a short period. The mice were arranged for blood sampling at 6 h, 12 h, 24 h, 3 d, 8 d, and 14 d post irradiation (5 animals from each group for each time point). We collected 50 µl blood from each individual by puncturing the mandibular vein with the Goldenrod lancet. The samples were kept in tubes coated with EDTA to prevent clotting and were then sent out for whole-blood counting.

### 2.4. Histological Processing

Tissues about 1 cm long were collected from three different intestinal parts (near-end, middle, and far-end) at 2 days post 16 Gy irradiation for immunofluorescence staining. The tissue was rinsed with 0.9% saline and embedded with an optimal cutting temperature (OCT) compound. Ten-micron-thick frozen sections were stained with Ki67 antibody. Only the sections with exceedingly reduced Ki67-positive cells were considered to belong to the irradiated groups. For the measurement of muscularis externa thickness, the intestinal tissue was collected at 280 days post 16.2 Gy irradiation. The tissue was fixed with formalin and rolled with the Swiss-rolling technique [28]. Following paraffin embedding, the Swiss roll was cut into 5 µm thick sections and stained with hematoxylin and eosin (H&E).

### 2.5. Statistical Analysis

Graphpad Prism 7 software [29] was used for the analysis. Three main endpoints were evaluated: the survival fraction, the number of proliferating crypt cells, and the depletion of immune cells in the peripheral blood. The comparison of crypt cell numbers and the depletion of the immune cells was conducted with one-way ANOVA. The log-rank test was used in the analysis of the survival data. All data are presented as mean ± SEM, and a *p*-value of *p* < 0.05 was considered to be statistically significant.

## 3. Results

### 3.1. Abdominal FLASH Irradiation Decreased Survival

**Figure 1:**
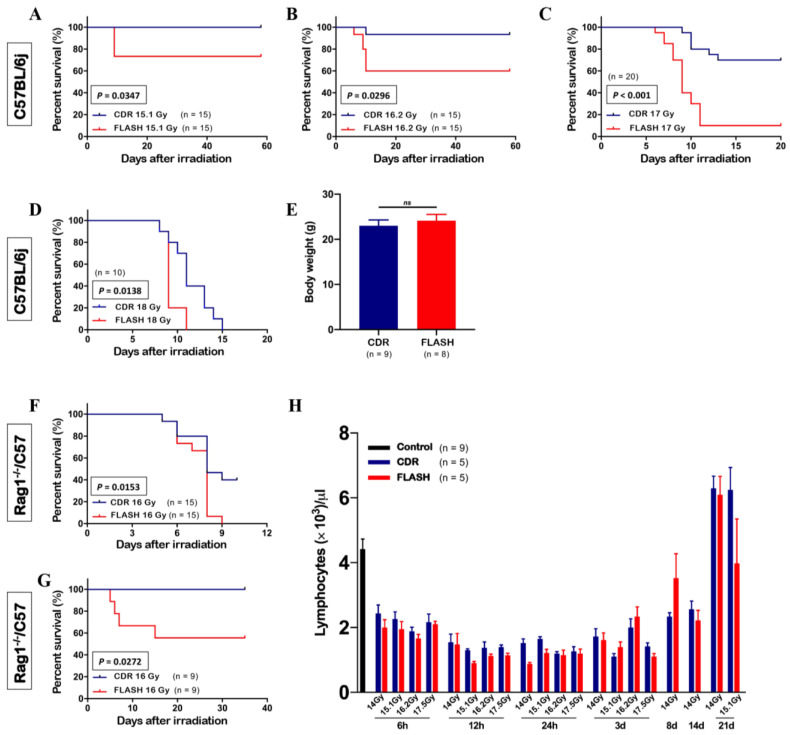
(**A**–**H**) Survival curves of C57BL/6j mice after FLASH and CDR irradiation at different dose levels: (**A**) 15.1 Gy; (**B**) 16.2 Gy; (**C**) 17 Gy; (**D**) 18 Gy. (**E**) Body weight of C57BL/6j mice at 280 days post exposure. (**F**,**G**) Survival curves of Rag1^−/−^/C57 mice with different body weights ((**F**) 18.8g; (**G**) 24.5 g) following 16 Gy irradiation. (**H**) Number of circulating lymphocytes in C57BL/6j mice at different timepoints. ns, not significant.

For C57BL/6j mice, partial abdominal irradiation of 14 Gy did not lead to any deaths independent of the dose rate. No mice died after 15.1 Gy CDR irradiation, while 4 out of 15 mice died following 15.1 Gy FLASH irradiation (*p* = 0.03) (Figure 1A). Compared to the corresponding CDR dose cohorts, 16.2 Gy (Figure 1B) and 17 Gy (Figure 1C) FLASH irradiation killed more mice within 2 weeks (*p* = 0.03 and *p* < 0.001, respectively). All of the mice died within 15 days after 18 Gy irradiation in both groups (Figure 1D). Another repeat experiment at 16.2 Gy showed no body weight difference between the FLASH and CDR groups at 280 days post exposure (Figure 1E). Independent of the dose rate, we found that if the mice survived the first 15 days, they recovered and lived long enough to reach the end of the study.

Rag1^−/−^/C57 mice, lacking mature B and T lymphocytes, showed no survival benefit following FLASH irradiation. Mice of different body weights had different percentages of abdominal volume exposed (due to their different sizes). FLASH irradiation caused more death in both 18.8 g (Figure 1F) and 24.5 g (Figure 1G) mice than in their counterparts in the CDR groups (*p* = 0.02 and *p* = 0.03, respectively).

### 3.2. FLASH Irradiation Did Not Preserve Circulating Lymphocytes

A lower percentage of circulating lymphocytes receiving irradiation due to the short irradiation time of FLASH irradiation was suggested by some publications [6,30] to be a mechanism underlying the FLASH effect. In the present study, whole-blood counting was performed to study the impact of dose rate on circulating-lymphocyte depletion. Compared to the non-irradiated group, the number of lymphocytes decreased dramatically during the first 24 h following exposure. The counts of lymphocytes showed a recovering trend on the third day. To reduce stress for the mice at higher dose levels, blood samples were only collected from the 14 Gy groups at 7, 14, and 21 days post irradiation and from the 15.1 Gy group on the 21st day. There was no significant difference in lymphocyte numbers between the FLASH and CDR cohorts at any timepoint. The number of lymphocytes returned to the baseline level at 21 days following exposure (Figure 1H).

### 3.3. Abdominal FLASH Irradiation Did Not Spare the Number of Proliferating Crypt Cells or the Muscularis Externa

**Figure 2:**
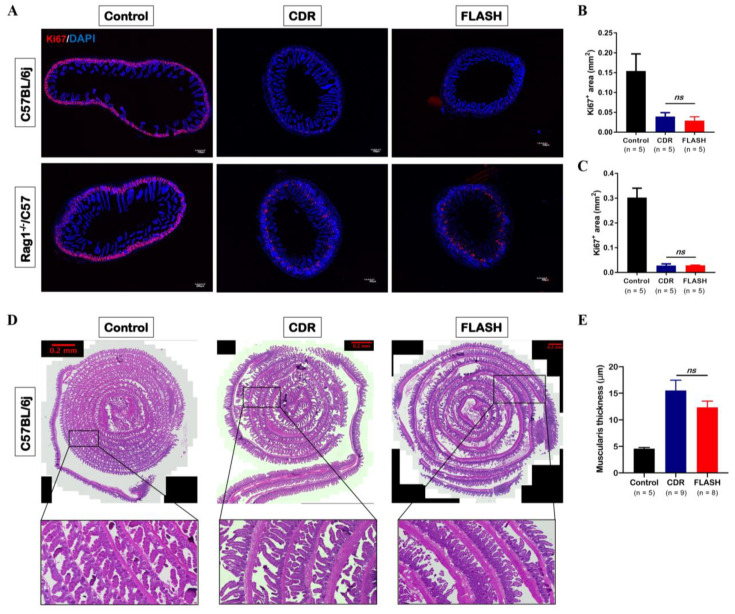
(**A**) Ki67 immunofluorescence staining images of intestinal tissues in C57BL/6j and Rag1^−/−^/C57 mice. (**B**) Quantification of the Ki67-positive area in C57BL/6j mice. (**C**) Quantification of the Ki67-positive area in Rag1^−/−^/C57 mice. (**D**) HE-stained images of a C57BL/6j mouse intestinal roll. (**E**) Quantification of muscularis externa thickness. ns, not significant.

The vast majority of the intestinal crypts were disrupted by 16 Gy irradiation. To reduce the subjective selection of specific crypts, the Ki67-positive (Ki67+) area of the intestine cross-section was measured. Compared with the control group, significantly fewer Ki67+ crypt cells were observed at 2 days following 16 Gy. FLASH and CDR irradiation killed similar numbers of proliferating crypt cells in both C57BL/6j and Rag1^−/−^/C57 mice (Figure 2A–C). Long-term follow-up showed significant thickening of the muscularis externa at 280 days after 16.2 Gy irradiation, with FLASH irradiation showing no statistically significant protection of the muscle layer compared to the CDR group (Figure 2D,E).

## 4. Discussion

One goal of the present study was to test the hypothesis that the FLASH effect is caused by the sparing of circulating lymphocytes due to the limited blood flow through the radiation field during FLASH irradiation. Mice with a normal vs. a compromised immune system were used. If the hypothesis is true, it should not be possible to observe a FLASH effect in Rag1^−/−^/C57 mice, which lack mature B and T cells. However, 120 Gy/s irradiation did not benefit the C57BL/6j mice. In fact, it resulted in more deaths in Rag1^−/−^/C57 mice, irrespective of body weight. Due to the absence of an observed FLASH effect, we cannot rule out circulating lymphocytes as a potential mechanism in cases where a FLASH effect is observed. One consideration is that only about 2% of whole-body lymphocytes are distributed in the blood [31,32]. Thus, a potential differential in the loss of circulating lymphocytes due to very short exposures may be overshadowed by the loss of lymphocytes in solid tissues such as the intestine and the spleen, which were partially in the radiation field and contain large fractions of lymphocytes. Due to the reliance on pre-irradiation imaging for positioning, we were not able to determine the position of all organs at the time of irradiation.

Our previous small-scale study including five mice per group showed a survival benefit after 16 Gy FLASH irradiation [27]. In that very preliminary study, three out of five mice in the CDR group died after 16 Gy irradiation, while all the mice in the FLASH group survived. As stated in that report, a different outcome of one mouse in either cohort would have led to non-significant results. In the present study, with a substantially larger sample size, we found that all endpoints, including the survival fraction of mice, the surviving proliferating crypt cells, and the counts of circulating lymphocytes showed no FLASH-induced tissue-sparing effect at any dose level. The only difference between the previous study and the present one is the irradiation procedure. In the previous study, the mice in the FLASH cohort were irradiated after the completion of all CDR irradiation. Alternate FLASH and CDR irradiations were conducted with every animal in the present study to exclude any potential time-related interference factors. However, this modification is not expected to influence the FLASH effect. Dosimetry was validated with the thin-gap ion chamber and thimble chamber before each independent experiment, and occasionally additional dosimetry was validated (as discussed previously) to ensure consistent and reproducible dosimetry. The recombination rate within our thin-gap ion chamber, which was used for online dosimetry, was less than 2% at maximum dose rate, which has also been reported previously [27]. Thus, dose uncertainties are an unlikely cause of the observed effects. The discrepant results between the initial and repeated studies highlight the importance of the reproducibility of FLASH experiments. Due to the high statistical power and diverse dose levels used in the present study, we conclude that FLASH does not reduce radiation-induced damage to the abdominal tissues under our exposure conditions.

Our group is not the only one that has observed a more pronounced depletion of lymphocytes following FLASH irradiation. Bhanu et al. reported that cardiac and splenic irradiation with a 20 MeV electron beam at a dose rate of 35 Gy/s killed more circulating lymphocytes than 0.1 Gy/s irradiation. In their study, a single fraction of 16 Gy whole abdominal irradiation induced 100% mortality within 7 days, while most of the mice survived after the same dose of conventional dose rate irradiation [12]. The authors ascribed the worse outcomes following FLASH irradiation to using an insufficiently high dose rate. Smyth et al. also reported a lack of sparing of normal tissue for photon irradiations of 37 to 41 Gy/s [33]. Although the results described above suggest no FLASH sparing effect on the gut, there are other publications that report a FLASH effect. Levy et al. demonstrated improved survival, spared gut function, and increased crypt cell survival post 16 Gy electron FLASH irradiation [14]. Ruan et al. found improvements in crypt survival and fewer changes to the microbiota after FLASH irradiation with a 6 MeV electron linear accelerator [34]. Ultra-high dose rate photon irradiation reportedly reduced intestinal pyroptosis and attenuated abdominal toxicity [35]. Less mortality was also observed following proton FLASH exposure when the mouse was placed either in the plateau region before the Bragg peak or within the spread-out Bragg peak (SOBP) [13,36]. The average dose rates of these studies were 216 Gy/s, 280 Gy/s, 110–120 Gy/s, 78 ± 9 Gy/s, and 100 Gy/s, respectively. Of note, proton, photon, and electron beams feature very different beam-pulse structures, which results in the instantaneous dose rates of beams, i.e., during the beam-on time, varying by orders of magnitude, with electron beams typically achieving the highest instantaneous dose rates. Proton beams using a cyclotron are delivered with a near-continuous beam structure; their pulse structure is only visible at the nanosecond scale. 

Diverse endpoints including survival fraction, weight loss, surviving intestinal crypts, proliferating cells per crypt, muscle layer thickness, and alteration of the gut microbiota were used in the reports on the abdominal FLASH effect. The first two endpoints tend to be less affected by artifacts, as far fewer experimental procedures are performed to collect the data of these two endpoints than of others.

The FLASH dose rate used in the present experiment was ~120 Gy/s, which is generally assumed to be high enough to induce a FLASH effect. Another study by our group using the same FLASH proton system showed a positive FLASH effect on mouse skin [37], which demonstrates the reliability of the experiment setup. The observed increase in deaths post 120 Gy/s FLASH irradiation suggests that other factors in addition to the mean dose rate determine the onset of the FLASH effect. It is not yet clear if physics-, chemistry-, or biology-related factors, or a combination of these, are the determining factors. To allow for future reanalysis of current studies, variables such as field size; fraction of the target organ within the field; and mouse-to-mouse variation in the 90 % isodose volume to the target, the pattern of pulses, the dose rate within each pulse, and the dose per pulse should also be reported (for details on our beam see Zhang et al. [27]). An interesting coincidence is that similar beam structures were generated by cyclotrons in the study by Diffenderfer et al. [13] and our study. The proton beams were generated with the same cyclotron system (IBA), and thus were delivered at the same radiofrequency of 106 MHz, and the beam pulses were alike (2 ns and 3 ns). Evans et al. [36] used a synchrocyclotron with a repetition rate of 756 Hz and a pulse time of 21 μs for their research. The most obvious difference between the experiments is the percentage of the abdomen that was irradiated. We conducted only partial abdominal irradiation in the present study, as the field size was limited to 16 × 12 mm^2^ to keep the dose rate above 100 Gy/s, whereas the reports by Diffenderfer et al. [13] and Evans et al. [36] used a 20 × 20 mm^2^ field size. About 60% of the whole-abdomen volume was irradiated in this scenario. The absence of a FLASH effect may result from distinct compensation mechanisms employed by unirradiated intestine. However, this does not explain why higher mortality was observed in the FLASH groups.

Overall, our results do not show a FLASH effect for partial-gut irradiation. Understanding the differences between our settings (partial gut, 120 Gy/s proton irradiations, 14–17 Gy) and the ones used at other institutes that showed FLASH tissue sparing in the gut (esp. with proton irradiations such as those by Diffenderfer et al. [13] and Evans et al. [36]) may help to determine the ideal FLASH tissue-sparing conditions.

## 5. Conclusions

FLASH proton irradiation did not spare intestinal tissue or circulating blood lymphocytes in the present study. To date, the reported in vivo experiments in which a FLASH effect has not been observed have involved abdominal irradiation. The diversity of abdominal organs included in the field makes it difficult to assess the FLASH effect solely on intestinal tissue. However, the results suggest that if a FLASH effect does pertain to several tissues, it does not pertain to all tissues, at least for the mean dose rate employed in this study. It is also possible, and perhaps likely, that total gut or total abdominal irradiation may elicit a FLASH effect, but not partial-gut irradiation. Careful investigations are needed to understand the conditions under which a FLASH effect may be observed. Understanding the limitations of FLASH is imperative for its clinical translation.

## Data Availability

Any data or materials that support the findings of this study can be made available by the corresponding author upon request.
